# MicroRNA-146a Promotes Embryonic Stem Cell Differentiation towards Vascular Smooth Muscle Cells through Regulation of Kruppel-like Factor 4

**DOI:** 10.1007/s11596-023-2736-3

**Published:** 2023-04-19

**Authors:** Qing Zhang, Rong-rong Pan, Yu-tao Wu, Yu-miao Wei

**Affiliations:** 1 Department of Cardiology, Union Hospital, Tongji Medical College, Huazhong University of Science and Technology, Wuhan, 430022 China; 2 Hubei Key Laboratory of Biological Targeted Therapy, Union Hospital, Tongji Medical College, Huazhong University of Science and Technology, Wuhan, 430022 China; 3 Hubei Provincial Engineering Research Center of Immunological Diagnosis and Therapy for Cardiovascular Diseases, Union Hospital, Tongji Medical College, Huazhong University of Science and Technology, Wuhan, 430022 China; 4 Department of Cardiology, Affiliated Cixi Hospital, Wenzhou Medical University, Ningbo, 315300 China; 5 Department of Cardiology, First Affiliated Hospital, School of Medicine, Zhejiang University, Hangzhou, 310003 China

**Keywords:** microRNA-146a, embryonic stem cells, differentiation, vascular smooth muscle cells, Kruppel-like factor 4

## Abstract

**Objective:**

Vascular smooth muscle cell (VSMC) differentiation from stem cells is one source of the increasing number of VSMCs that are involved in vascular remodeling-related diseases such as hypertension, atherosclerosis, and restenosis. MicroRNA-146a (miR-146a) has been proven to be involved in cell proliferation, migration, and tumor metabolism. However, little is known about the functional role of miR-146a in VSMC differentiation from embryonic stem cells (ESCs). This study aimed to determine the role of miR-146a in VSMC differentiation from ESCs.

**Methods:**

Mouse ESCs were differentiated into VSMCs, and the cell extracts were analyzed by Western blotting and RT-qPCR. In addition, luciferase reporter assays using ESCs transfected with miR-146a/mimic and plasmids were performed. Finally, C57BL/6J female mice were injected with mimic or miR-146a-overexpressing ESCs, and immunohistochemistry, Western blotting, and RT-qPCR assays were carried out on tissue samples from these mice.

**Results:**

miR-146a was significantly upregulated during VSMC differentiation, accompanied with the VSMC-specific marker genes smooth muscle-alpha-actin (SMαA), smooth muscle 22 (SM22), smooth muscle myosin heavy chain (SMMHC), and h1-calponin. Furthermore, overexpression of miR-146a enhanced the differentiation process *in vitro* and *in vivo*. Concurrently, the expression of Kruppel-like factor 4 (KLF4), predicted as one of the top targets of miR-146a, was sharply decreased in miR-146a-overexpressing ESCs. Importantly, inhibiting KLF4 expression enhanced the VSMC-specific gene expression induced by miR-146a overexpression in differentiating ESCs. In addition, miR-146a upregulated the mRNA expression levels and transcriptional activity of VSMC differentiation-related transcription factors, including serum response factor (SRF) and myocyte enhancer factor 2c (MEF-2c).

**Conclusion:**

Our data support that miR-146a promotes ESC-VSMC differentiation through regulating KLF4 and modulating the transcription factor activity of VSMCs.

**Electronic supplementary material:**

The online version of this article (10.1007/s11596-023-2736-3) contains supplementary material, which is available to authorized users.

Vascular smooth muscle cells (VSMCs) are the major component of the vascular wall and play an important role in the formation and progression of atherosclerotic plaques and other cardiovascular diseases, such as restenosis and hypertension[1]. Understanding the transcriptional regulatory circuitry of VSMC differentiation is essential to prevent these diseases and may prove useful for developing stem cell therapies.

Qing ZHANG, E-mail: 21718081@zju.edu.cn; Rong-rong PAN, E-mail: panrong_0131@126.com
†These authors contributed equally to this work.
#Corresponding authors, Yu-tao WU, E-mail: banshuirentaotao@126.com; Yu-miao WEI, E-mail: ymwei12@163.com
*This work was funded by the National Natural Science Foundation of China (No. 82070376 and No. 81873491), the Natural Science Foundation of Zhejiang Province (No. LY21H020005), the Zhejiang Medical Science and Technology Project (No. 2019KY376 and No. 2018KY071), and a Ningbo Science and Technology Project (No. 202002N3173).
**Electronic supplementary material** The online version of this article (https://doi.org/10.1007/s11596-023-2736-3) contains supplementary material, which is available to authorized users.

MicroRNAs (miRs) are small noncoding sequences of RNAs of about 22 nucleotides in length that have been implicated recently in embryonic development, stem cell differentiation, and other physiological and pathological processes[2, 3]. Mechanistically, miRs function as antisense regulators of their target genes by binding to the 3′-untranslated region (UTR) of mRNAs and regulating translation. Multiple miRs have been reported to play critical roles in self-renewal and differentiation programs of stem cells involved in vascular remodeling-related diseases. The miR-199a/b, miR-200a, miR-155, and miR-21 have shown major roles in regulating endothelial cell differentiation from stem cells[4–8]; while other miRs, such as miR-22, miR-34, miR-214, and miR-133, have been identified as key factors in regulating VSMC function[9–12]. Our previous studies have shown that miRs, including miR-22, miR-34, miR-29, and miR-214, also play essential roles in regulating VSMC differentiation from stem cells[13–16].

MiR-146a has been reported to be involved in inflammatory and immune responses as well as cell proliferation, migration, and apoptosis[17–20]. It was first reported to negatively regulate the interleukin-1beta-induced inflammatory response in human lung alveolar epithelial cells[21]. In addition, miR-146 has been demonstrated to inhibit nuclear factor-kappa B activity by reducing the metastatic potential of breast cancer cells[22]. Moreover, Yuan *et al*[23] have revealed that miR-146a modulates the transforming growth factor-beta 1-induced phenotypic differentiation of human dermal fibroblasts. Furthermore, there have been studies investigating the role of miR-146a in other cardiovascular diseases such as myocardial ischemia/reperfusion injury and cardiac hypertrophy/dysfunction[24, 25]. However, little is known about the functional involvement of miR-146a in VSMC differentiation from embryonic stem cells (ESCs). Therefore, this study aimed to determine the role of miR-146a in VSMC differentiation from ESCs.

## 1 MATERIALS AND METHODS

### 1.1 Cell Culture and Differentiation

Detailed protocols for culture of mouse ESCs (ES-D3 cell line, CRL-1934; ATCC, USA) and VSMC differentiation are described in our previous studies[13, 26]. Briefly, ESCs were dispersed on gelatin (G1393, Sigma, USA)-coated flasks and cultured in culture medium including knockout Dulbecco's Modified Essential Medium (Gibco, USA), 10% fetal bovine serum (FBS, 10099141, Gibco, USA), 10 ng/mL leukemia inhibitor factor (ESG1107, Millipore, USA), 0.1 mmol/L 2-mercaptoethanol (2-ME, M6250, Sigma, USA), 100 U/mL penicillin, 100 μg/mL streptomycin (15140155, Invitrogen, USA), and 2 mmol/L glutamine (G3126, Sigma-Aldrich, USA). The cells were split every 3 days at a ratio of 1:4. For VSMC differentiation, undifferentiated ESCs were seeded on mouse collagen Ⅳ (5 μg/mL, 356233l, Corning, USA)-coated flasks in differentiation medium (DM) that contained alpha-minimal essential medium (41061-029, Gibco, USA) supplemented with 10% FBS, 0.05 mmol/L 2-ME, 100 U/mL penicillin, 100 μg/mL streptomycin, and 2 mmol/L glutamine. The DM was refreshed every day after the second day of differentiation. The cells were cultured in DM for 4–8 days before being harvested for further analysis.

### 1.2 Western Blotting Analysis

The detailed procedure was similar to that described in our previous studies[13, 26]. Briefly, cells were harvested and lysed with RIPA (P0013B, Beyotime, China) supplemented with protease inhibitors (P105539-5g, Aladdin, China). The total protein was quantified using a BCA Protein Assay Kit (P0012S, Beyotime, China). Equal amounts of protein boiled for 10 min (100°C) in 5× SDS loading buffer (P0015, Beyotime, China) were resolved by SDS-PAGE in a 5%–12% tris-glycine gel. The proteins were transferred from the gel to a membrane and blocked with 5% skim milk for 1 h at room temperature. The membrane was incubated with primary antibodies, including antibodies against GAPDH (14C10, CST, USA), smooth muscle alpha-actin (SMαA), 14395-1-AP (Proteintech, USA), smooth muscle myosin heavy chain (SMMHC) (AHP1117, AbD Serotec, UK), smooth muscle 22 (SM22) (Ab14106, Abcam, UK), and h1-calponin (Ab78491, Abcam, UK), respectively. All secondary antibodies were acquired from Proteintech (USA). The GAPDH protein was used as an internal control to ensure equal amounts of protein loading among the groups, and the results were subjected to standard Western blotting analysis.

### 1.3 RT-qPCR for mRNA and miRNA

RT-qPCR was performed as described previously[27]. Briefly, RNA was extracted from the cells using Trizol (12183555, Invitrogen, USA) according to the manufacturer's instructions. Total RNA- and miRNA-specific cDNA syntheses were performed with a PrimeScript RT Master Mix (Perfect Real Time) Kit (RR047A, Takara Bio, Japan) and an miDETECT A Track™ miRNA RT-qPCR Starter Kit (C10712-1, Ribobio, China), separately. The RT-qPCR was run on an ABI Prism 7500 system, using Takara premix Ex Taq Ⅱ (DRR820A, Takara, Japan) or an miDETECT A TrackTM miRNA RT-qPCR Starter Kit. The primers used in this study are listed in table 1. The relative mRNA/microRNA expression level was defined as the ratio to the 18S/U6 expression level, with that of the control sample set as 1.0.

### 1.4 miRNA, siRNA, and Plasmid Transfection

When cultured cells reached 60%–70% confluence, the media were replaced with 1 mL of

**Table 1 RT-qPCR primers used in the present study**

| Gene name | Forward (5′–3′) | Reverse (5′–3′) |
|---|---|---|
| Mmu-18S rRNA | AAACGGCTACCACATCCAG | CCTCCAATGGATCCTCGTTA |
| Mmu-U6 snRNA | GATGACACGCAAATTCGTG | miRNA universal reverse primer |
| Mmu-miR-146a | CGGTGAGAACTGAATTCCATGGGTT | miRNA universal reverse primer |
| Mmu-KLF4 | GTGCCCCGACTAACCGTTG | GTCGTTGAACTCCTCGGTCT |
| Mmu-SMαA | TCCTGACGCTGAAGTATCCGAT | GGCCACACGAAGCTCGTTATAG |
| Mmu-SM22 | GATATGGCAGCAGTGCAGAG | AGTTGGCTGTCTGTGAAGTC |
| Mmu-h1-calponin | GGTCCTGCCTACGGCTTGTC | TCGCAAAGAATGATCCCGTC |
| Mmu-SMMHC | AAGCAGCCAGCATCAAGGAG | AGCTCTGCCATGTCCTCCAC |
| Mmu-SRF | CCTACCAGGTGTCGGAATCTGA | TCTGGATTGTGGAGGTGGTACC |
| Mmu-MEF-2c | AAGCCAAATCTCCTCCCCCTAT | TGATTCACTGATGGCATCGTGT |
| Mmu-SKI | CCGCAGATCCTCAACTCGG | GCGTCCGTCTTAGTGATGAGC |
| Mmu-YY1 | CAGTGGTTGAAGAGCAGATCAT | AGGGAGTTTCTTGCCTGTCAT |
| Mmu-Sirt1 | ATGACGCTGTGGCAGATTGTT | CCGCAAGGCGAGCATAGAT |
| Mmu-E2f3 | AAACGCGGTATGATACGTCCC | CCATCAGGAGACTGGCTCAG |
| Mmu-SP1 | AGGGTCCGAGTCAGTCAGG | CTCGCTGCCATTGGTACTGTT |
| Mmu-SMAD2 | ATGTCGTCCATCTTGCCATTC | AACCGTCCTGTTTTCTTTAGCTT |
| Mmu-SMAD3 | CACGCAGAACGTGAACACC | GGCAGTAGATAACGTGAGGGA |
| Mmu-QKI | CTGGACGAAGAAATTAGCAGAGT | ACTGCCATTTAACGTGTCATTGT |
| Mmu-Notch1 | ACTTGTCAGATGTGGCCTCG | ATTCAAGTGGCTGATGCCCA |

empty media without antibiotics and maintained for 30 min before transfection. The miRNA, siRNA, or plasmid (BAIAO, China) was transfected into the ESCs using Lipofectamine 3000 (L3000-015, Invitrogen, USA) according to the manufacturer's protocol. The transfected cells were cultured for 48–96 h in DM to allow VSMC differentiation. miR-146a/mimic and siRNAs (normal control or siRNA-KLF4) were cotransfected using jetPRIME® (Polyplus SA, France).

### 1.5 Luciferase Reporter Assay

Luciferase reporter assays were performed as described in our previous studies[27]. A total of $6×10^4$ ESCs were seeded in DM in a collagen-coated 12-well plate. After 96 h, the cells were cotransfected with the luciferase reporter plasmids [serum response factor (SRF) and myocyte-specific enhancer factor-2c (MEF-2c), 0.33 μg/well] and miR-146a, miR-146a inhibitor, or control (100 nmol/L) using Lipofectamine 3000. *Renilla* (0.1 μg/well, Promega, USA) was included in all transfection assays as an internal control. Luciferase and *Renilla* activity assays were performed 48 h after transfection. The relative luciferase unit was defined as the ratio of luciferase activity to *Renilla* activity. The value of the control was set as 1.0.

### 1.6 Animal Study

The detailed procedure for the animal studies was very similar to that described previously[14]. All animal experiments were conducted according to the ARRIVE guidelines. All animal procedures were carried out in accordance with the provisions of the Declaration of Helsinki (as revised in Edinburgh in 2000) and were approved by the Research Ethics Committee of the First Affiliated Hospital of Zhejiang University (Institutional Review Board Approval No. 2013/150). Briefly, C57BL/6J female mice were injected with mimic- or miR-146a-overexpressing ESCs ($10^6$ cells) mixed with 50 μL of Matrigel. After 10–13 days, the mice were sacrificed and the implanted Matrigel plugs were harvested for SMαA or SMMHC immunofluorescence staining. Partial Matrigel implants were used to extract the total RNA and protein samples for RT-qPCR and Western blotting analysis, respectively.

### 1.7 Statistical Analysis

For all animal experiments, at least 6 animals were used ($n≥6$); while the cell experiments were performed with at least 3 samples ($n≥3$). Independent repeated experiments were performed at least in triplicate. All data were checked before analysis to confirm that the data conformed to a normal distribution. The data were expressed as the mean±standard error of mean (SEM) and analyzed using a two-tailed Student's *t*-test for two-group comparison. Comparisons of different groups were performed using one-way ANOVA followed by Tukey's HSD multiple comparison post-hoc test. A value of $P<0.05$ was considered statistically significant.

## 2 RESULTS

### 2.1 miR-146a Mediated VSMC Differentiation from ESCs *In Vitro*

To induce VSMC differentiation, ESCs were seeded on collagen Ⅳ-coated 6-well plates and cultured in VSMC DM for 6 days. Consistently, VSMC-specific markers, including SMαA (fig. 1A), SM22 (fig. 1B), and SMMHC (fig. 1C), were significantly upregulated during differentiation. Simultaneously, miR-146a was significantly upregulated during VSMC differentiation (fig. 1D), suggesting a significant role

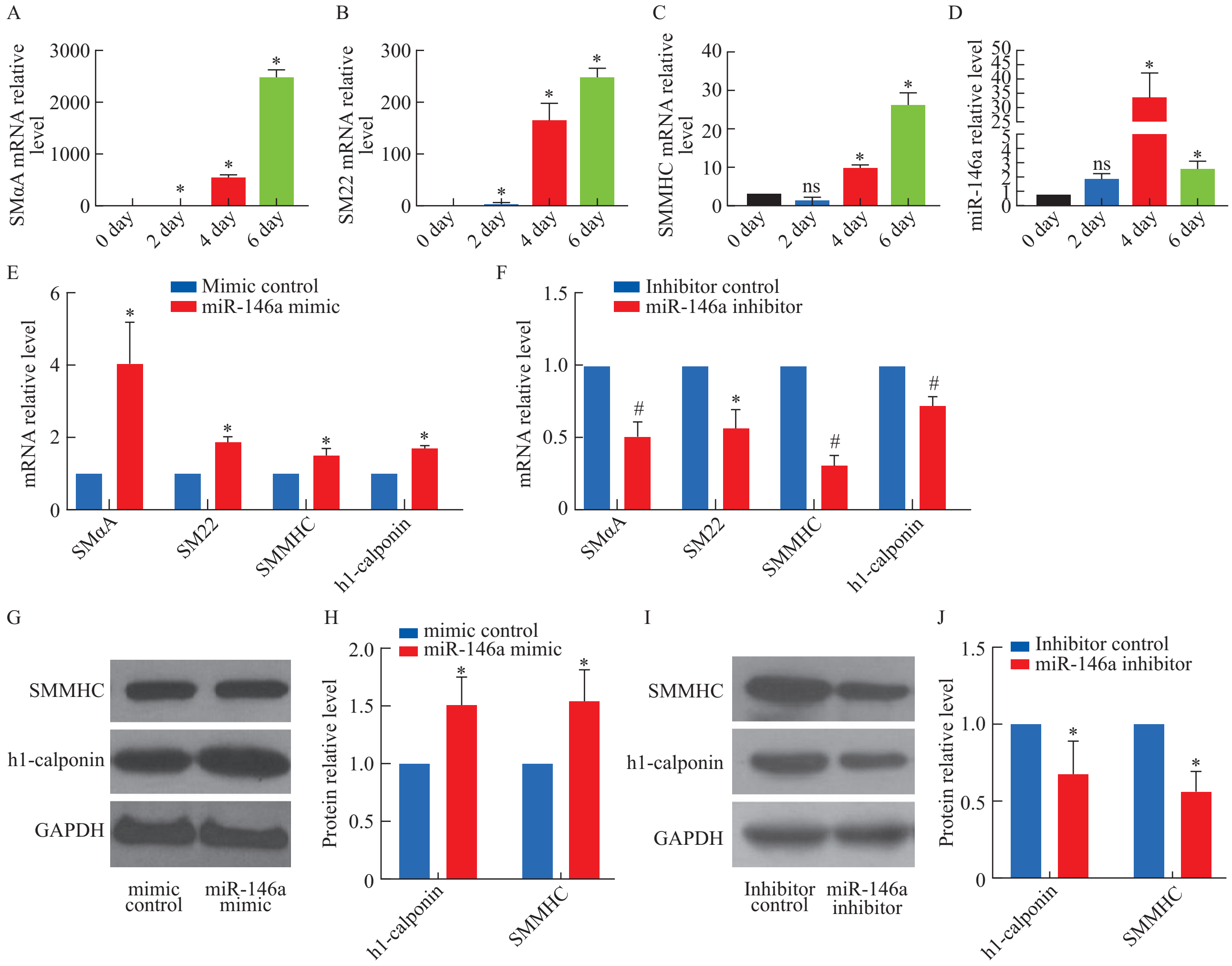

**Fig. 1** miR-146a promoted mouse embryonic stem cell (ESC) differentiation towards vascular smooth muscle cells (VSMCs)
ESCs were cultured on collagen Ⅳ-coated 6-well plates to allow ESC differentiation for 2, 4, and 6 days. A–C: VSMC-specific marker genes, including SMαA (A), SM22 (B), and SMMHC (C), were measured to determine ESC differentiation. D: During this process, miR-146a was significantly upregulated. Differentiating ESCs at day 3 were transfected with either miRNA mimic negative control (mimic control in short), miR-146a mimic, miRNA inhibitor negative control (inhibitor control in short), or miR-146a inhibitor. At 48–72 h after transfection, transfected cells were harvested and subjected to RT-qPCR (E–F) and Western blotting (G–J) analyses. The 0-day samples were undifferentiated ESCs and served as the negative control. The data are represented as the mean±SEM of at least three repeats. $^*P$<0.05 *vs*. control group for miR-146a during VSMC differentiation. Gain-of-function experiments using an miR-146a mimic showed that both the mRNA and protein levels (fig. 1E, 1G–1H) of smooth muscle differentiation-specific markers (SMαA, SM22, SMMHC, and h1-calponin) were significantly increased. Meanwhile, miR-146a inhibitor significantly inhibited the mRNA and protein expression of VSMC-specific markers (fig. 1F, 1I–1J), suggesting a critical *in-vitro* role of miR-146a in VSMC differentiation from ESCs.

### 2.2 miR-146a Promoted VSMC Differentiation *In Vivo*

To investigate whether miR-146a would promote VSMC differentiation *in vivo*, Matrigel plugs implanted with ESCs with or without miR-146a overexpression were injected subcutaneously into mice. As expected, more SMαA-positive and SMMHC-positive cells were observed in the implants of miR-146a-overexpressing ESCs (fig. 2A and 2B), and fewer SMαA-positive and SMMHC-positive cells were presented in the implants of miR-146a inhibitors (fig. S1A and S1B), as demonstrated by immunofluorescence staining and further ImageJ analysis (fig. 2C and 2D). In addition, we found that the miR-146a-overexpression implants had a higher expression of smooth muscle differentiation-specific markers, including SMαA, SM22, and SMMHC at the gene level (fig. 2E) and h1-calponin at the protein level (fig. 2F), further confirming the efficiency of miR-146a overexpression and the importance of miR-146a in VSMC differentiation *in vivo*. Taken together, these data firmly suggest a regulatory role of miR-146a in VSMC differentiation from ESCs both *in vitro* and *in vivo*.

### 2.3 miR-146a Promoted VSMC Differentiation Through Regulation of KLF4

To determine the target gene of miR-146a during

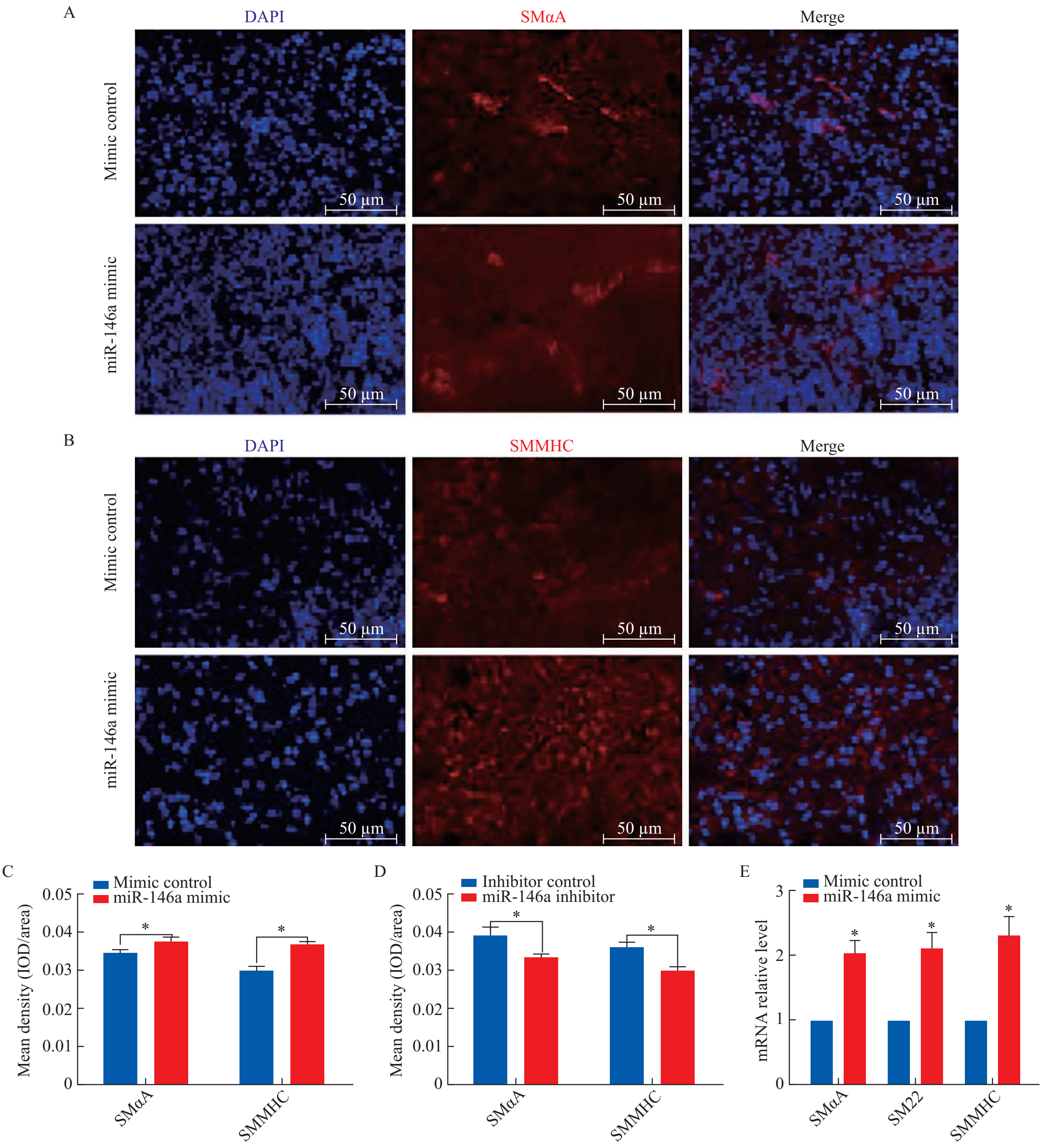

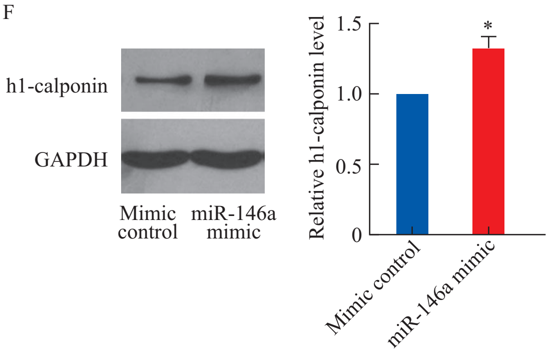

**Fig. 2** miR-146a positively modulated *in-vivo* VSMC differentiation from ESCs

Matrigel plugs implanted with ESCs without (mimic control) or with (miR-146a mimic) miR-146a overexpression were injected subcutaneously into mice. The plugs were harvested at 10th day and were sectioned and subjected to immunofluorescence staining using antibodies against SMαA/SMMHC (blue is DAPI, and red is SMαA/SMMHC). Some immunofluorescence staining data from Matrigel plugs implanted with ESCs without (inhibitor control) or with (miR-146a inhibitor) miR-146a inhibition are in the supplementary materials. A and B: Matrigel plugs with ESCs transfected with miR-146a showed more SMαA/SMMHC-positive cells. C and D: The mean density of SMαA/SMMHC immunofluorescence staining was measured by 3 well-skilled researchers. E and F: Total RNA and protein for RT-qPCR and Western blotting were extracted from Matrigel implants, according to the manufacturer's instructions. The data are represented as the mean±SEM of at least 3 repeats. $^*P<0.05$ *vs*. control group

VSMC differentiation, we tested the expression of potential target genes mentioned in previous studies[28] and according to the TargetScan MicroRNA targeting program (http://www. targetscan.org). We found that *KLF4* gene expression was significantly downregulated by miR-146a and displayed a negative correlation with the gene expression levels of miR-146a during VSMC differentiation (fig. 3A and 3B). These results suggested that KLF4 may be negatively regulated by miR-146a. Based on TargetScan analysis, miR-146a had complementary sequences to the 3′UTR of KLF4's mRNA (fig. 3C). Our data from the KLF4 inhibition experiment showed that siR-KLF4 significantly downregulated the expression of KLF4 during VSMC differentiation (fig. 3D), resulting in the upregulation of smooth muscle differentiation-specific markers, including SM22 and SMMHC (fig. 3E), revealing that knockdown of KLF4 expression may recapitulate the effects of miR-146a during VSMC differentiation. We further investigated whether KLF4 repression is required for miR-146a-mediated VSMC differentiation. Data from the miR-146a mimic and siR-KLF4 cotransfection assay showed that siR-KLF4 exacerbated the promotion of miR-146a expression on VSMC differentiation, which is characterized by the upregulated expression of SM22 and SMMHC (fig. 3F). From these data, we surmised that miR-146a promoted VSMC differentiation through regulation of KLF4.

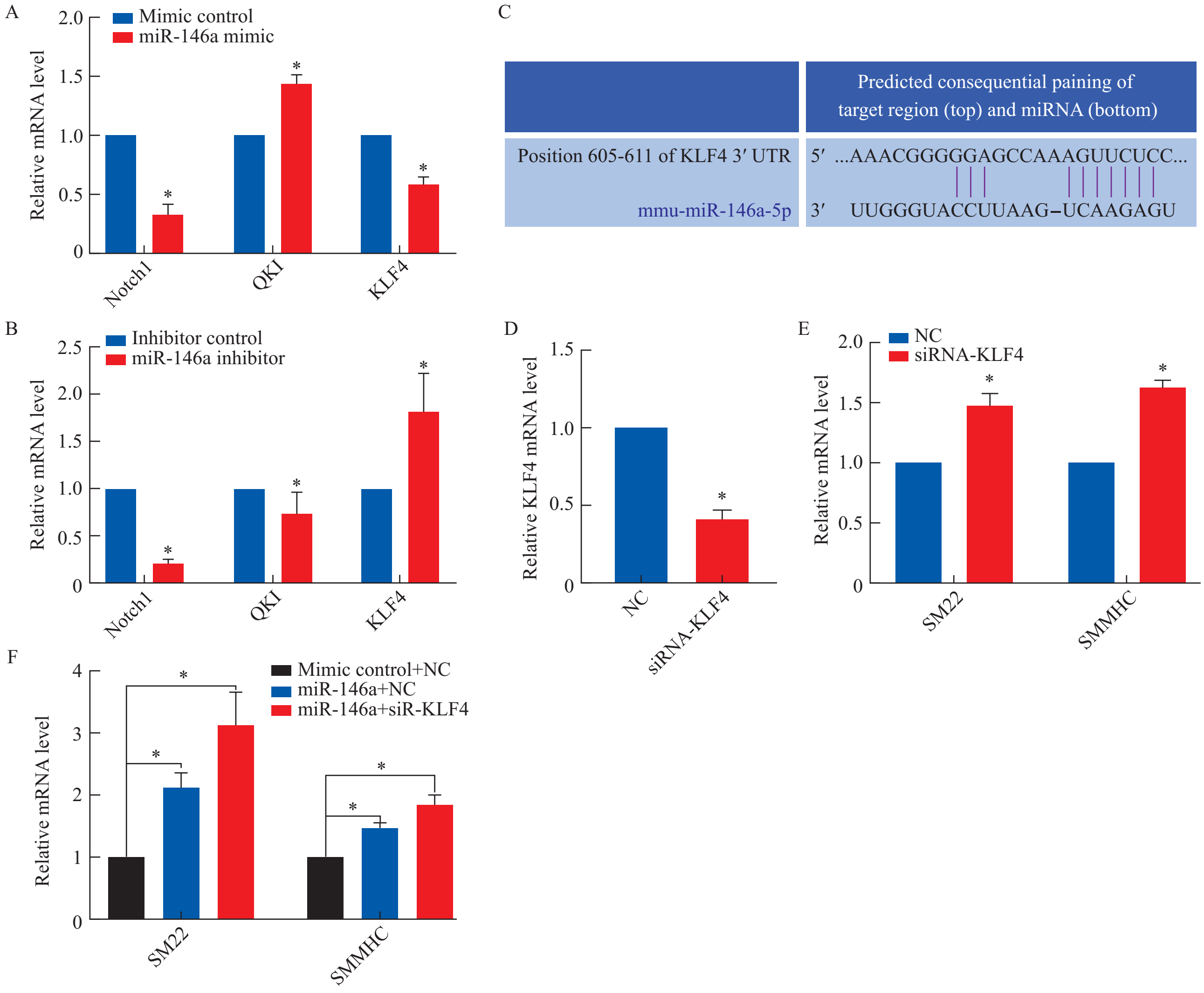

**Fig. 3** KLF4 may act as a VSMC differentiation repressor, and knockdown of KLF4 enhanced the promotive effect of miR-146a on ESC differentiation towards VSMCs

To detect the target gene of miR-146a during VSMC differentiation, the RNA of 3-day differentiating ESCs transfected by miR-146a mimic or miR-146a inhibitor was extracted for RT-qPCR. A and B: the expression of some potential target genes related to VSMC differentiation. C: the potential binding site of miR-146a within the 3′UTR of KLF4 as predicted by Targetscan. D and E: Differentiating ESCs from days 2–3 were transfected with normal control (NC) or siRNA-KLF4 and cultured in VSMC differentiation medium for another 48 or 72 h. Total RNA was extracted and subjected to RT-qPCR analyses. mRNA of KLF4 was downregulated by siRNA-KLF4, resulting in upregulation of the mRNA level of VSMC marker genes. F: Knockdown of KLF4 enhanced miR-146a-induced upregulation of the mRNA level of VSMC-specific genes. The data are represented as the mean±SEM of at least 3 repeats. $^{*}P$<0.05 *vs*. control group

### 2.4 Regulation of miR-146a-on VSMC Differentiation Possibly through Transcription Factors

SRF and MEF-2c are well-known transcription factors for VSMC gene expression regulation. We tested the transfection efficiency of the miR-146a mimic and the miR-146a inhibitor (fig. 4A and 4B), and the data showed that the gene expression levels of SRF and MEF-2c were significantly upregulated by the miR-146a mimic (fig. 4C) and downregulated by the miR-146a inhibitor (fig. 4D), suggesting that miR-146a may play a role in regulating these transcription factors during VSMC differentiation. Additionally, luciferase activity assays with SRF gene reporters also showed that the SRF gene promoter activities were related to the expression of miR-146a (fig. 4E and 4F), indicating that miR-146a upregulated the transcriptional activity of these genes.

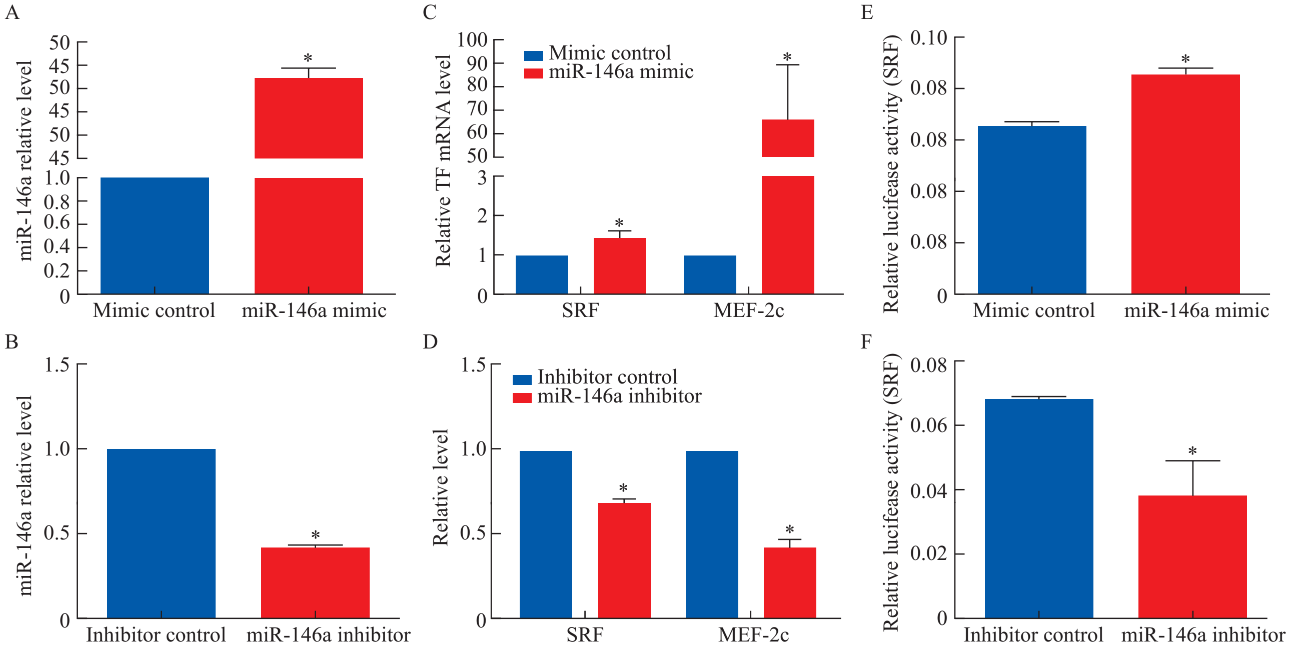

**Fig. 4** The expression of VSMC differentiation-related transcription factors (TFs) were positively modulated by miR-146a

Differentiating ESCs at day 3 were transfected with miR-146a mimic or miR-146a inhibitor and its control, and cultured in differentiation medium for 24 h. The transfected cells were harvested for RT-qPCR. A–D: The expression levels of miR-146a (A), serum response factor (SRF) (C), and myocyte-specific enhancer factor 2c (MEF-2c) (C) were significantly upregulated by miR-146a overexpression, while miR-146a inhibitor reduced the mRNA expression levels of miR-146a (B), SRF (D), and MEF-2c (D). E and F: The promoter activities of SRF were enhanced (E) or inbibited (F) by miR-146a mimic/miR-146a inhibitor. Day-3 differentiating ESCs seeded on a 24-well plate were transfected with the luciferase reporter plasmid pGL3-SRF-Luc (0.25 μg/well) together with miR-146a mimic or miR-146a inhibitor and its control (25 pmol/well). The plasmid *Renilla* (50 ng/well) was included as a control. Luciferase activity assays were performed 48 h after transfection. The data are represented as the mean±SEM of at least 3 repeats. $^{*}P<0.05$ *vs*. control group

## 3 DISCUSSION

VSMCs constitute an important component of blood vessels and carry out the physiological functions of contraction and relaxation of vessels, regulation of blood pressure, and blood flow distribution. Therefore, fully understanding the molecular mechanism of VSMC differentiation is essential in stem cell therapy research for vascular diseases and regenerative medicine. Accumulating evidence has demonstrated that mechanical forces, contractile agonists, extracellular matrix components, and other factors, including reactive oxygen species, Notch family members, and miRs, are responsible for VSMC differentiation. However, the mechanisms that regulate ESC–VSMC differentiation have not been fully elucidated. In the present study, we revealed an important role of miR-146a in regulating VSMC-specific gene expression *in vitro* and *in vivo* during VSMC differentiation from ESCs. We provided evidence to support that the identified target gene, *KLF4*, functions as an important gene repressor during VSMC differentiation from stem cells.

miR-146a has been investigated in different fields, such as inflammatory response, cancer cell migration, and angiogenesis[29–31]. We found that miR-146a was upregulated during ESC differentiation towards VSMCs, which was demonstrated by RT-qPCR, in accordance with the rise of miR-34a, miR-29a, and miR-214 levels[13–15]. As expected, we confirmed a critical role for miR-146a in VSMC differentiation from ESCs *in vitro*, using miRNA gain/loss-of-function analyses. Furthermore, by generating miR-146a-overexpression ESC lines and using our well-established *in-vivo* VSMC differentiation model, we provide evidence to support that miR-146a plays an important role in VSMC differentiation *in vivo*. These data confirm that miR-146a is an important VSMC differentiation regulator.

One of the findings of the current study is that we identified *KLF4* as the possible target gene of miR-146a during VSMC differentiation. miR-146a has been reported to inhibit glioma development by targeting Notch1[32], which is closely related to vascular biology and differentiation. Other target genes, such as *YY1*, *SP1*, and *Sirt1*, have been reported to be involved in VSMC differentiation by our group[13–15, 33, 34]. Here, we chose these genes as potential targets during VSMC differentiation. According to TargetScan and the RT-qPCR results (fig. 3A and 3B, fig. S2A and S2B), KLF4 was selected to be the potential target during the ESC differentiation model, which was demonstrated to be a target gene in miR-146a-induced VSMC proliferation[28]. Moreover, our results suggest that KLF4 was negatively regulated by miR-146a and KLF4 siRNA enhanced VSMC marker gene activation induced by miR-146a overexpression, supporting an important role of KLF4 in miR-146a-mediated VSMC differentiation. In addition, we demonstrated that miR-146a regulates the mRNA levels of key transcription factors (SRF and MEF-2c) and promoter activities in VSMCs. Furthermore, several studies have suggested the potential interplay between KLF4 and SRF in the transcriptional regulation of the *SMMHC* and *SM22* genes[35]. Taken together, miR-146a may promote VSMC differentiation from ESCs by attenuating KLF4's negative regulation to VSMC differentiation-specific transcription factors. However, the detailed relationships between KLF4 and these transcription factors were not fully covered in our study.

**Open Access**

This article is licensed under a Creative Commons Attribution 4.0 International License https://creativecommons.org/licenses/by/4.0/), which permits use, sharing, adaptation, distribution and reproduction in any medium or format, as long as you give appropriate credit to the original author(s) and the source, provide a link to the Creative Commons licence, and indicate if changes were made. The images or other third party material in this article are included in the article's Creative Commons licence, unless indicated otherwise in a credit line to the material. If material is not included in the article's Creative Commons licence and your intended use is not permitted by statutory regulation or exceeds the permitted use, you will need to obtain permission directly from the copyright holder. To view a copy of this licence, visit http://creativecommons.org/licenses/by/4.0/.

**Conflict of Interest Statement**

The authors declare that they have no conflicts of interest.

## Supplementary data

Appendix
