# Supplementary material for: MicroRNA-146a Promotes Embryonic Stem Cell Differentiation towards Vascular Smooth Muscle Cells through Regulation of Kruppel-like Factor 4
Source: Curr Med Sci. 2023 Apr 19;43(2):223–31. doi: 10.1007/s11596-023-2736-3 (PMC10112997; doi:10.1007/s11596-023-2736-3)
Supplement: Supplementary file 1 — Appendix [file 11596_2023_2736_MOESM1_ESM.pdf]

Current Medical Science
DOI https://doi.org/10.1007/s11596-023-2736-3
43(2),2023

Supplementary data

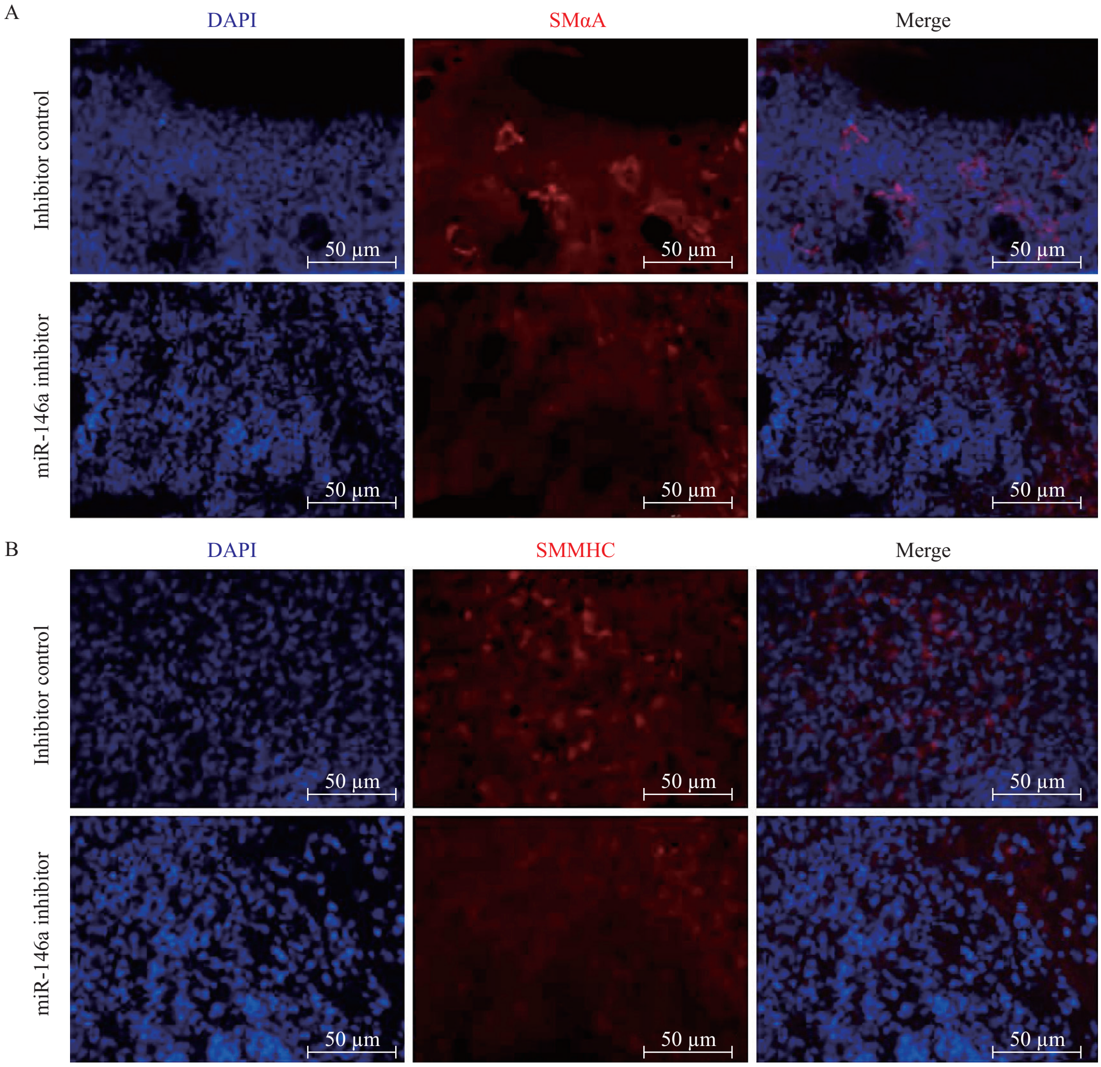

Fig. S1 Downregulation of miR-146a inhibits VSMCs differentiation from ESCs *in vivo*

A and B: Matrigel plugs implanted with ESCs without (inhibitor control) or with (miR-146a inhibitor) miR-146a inhibition were injected subcutaneously into mice. The plugs were harvested at 10th day and were sectioned and subjected to immunofluorescent staining using antibodies against SMαA/SMMHC. ESCs: embryonic stem cells; SMαA: smooth muscle-actin; SMMHC: smooth muscle myosin heavy chain; VSMCs: Vascular smooth muscle cells

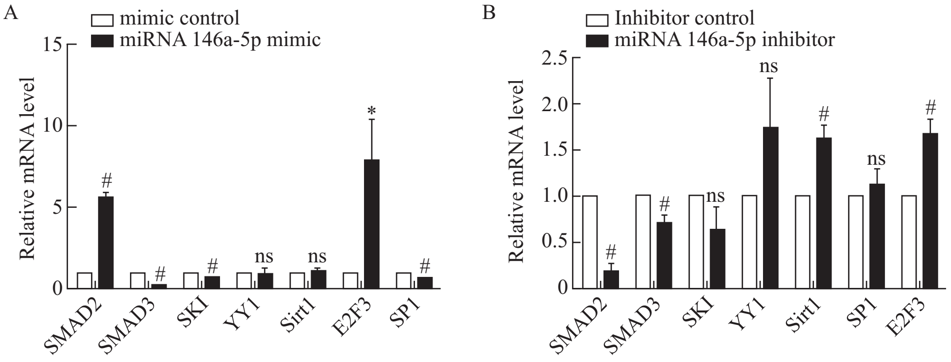

Fig. S2 Expression of some potential target genes of miR-146a

RNAs of 3-day differentiating ESCs transfected by miR-146a mimic (A) or miR-146a inhibitor (B) was extracted for RT-qPCR. $^{*}P$<0.05, $^{\#}P$<0.01. ESCs: embryonic stem cells
